# Active maintenance of eligibility trace in rodent prefrontal cortex

**DOI:** 10.1038/s41598-020-75820-0

**Published:** 2020-11-02

**Authors:** Dong-Hyun Lim, Young Ju Yoon, Eunsil Her, Suehee Huh, Min Whan Jung

**Affiliations:** 1 Department of Biological Sciences, Korea Advanced Institute of Science and Technology, Daejeon, 34141 Korea; 2 Center for Synaptic Brain Dysfunctions, Institute for Basic Science, Daejeon, 34141 Korea; 3 Department of Psychiatry, Ajou University School of Medicine, Suwon, 16499 Korea

**Keywords:** Learning and memory, Reward

## Abstract

Even though persistent neural activity has been proposed as a mechanism for maintaining eligibility trace, direct empirical evidence for active maintenance of eligibility trace has been lacking. We recorded neuronal activity in the medial prefrontal cortex (mPFC) in rats performing a dynamic foraging task in which a choice must be remembered until its outcome on the timescale of seconds for correct credit assignment. We found that mPFC neurons maintain significant choice signals during the time period between action selection and choice outcome. We also found that neural signals for choice, outcome, and action value converge in the mPFC when choice outcome was revealed. Our results indicate that the mPFC maintains choice signals necessary for temporal credit assignment in the form of persistent neural activity in our task. They also suggest that the mPFC might update action value by combining actively maintained eligibility trace with action value and outcome signals.

## Introduction

Making a causal link between an action and its outcome can be trivial when sensory information about the chosen action is available at the time its outcome is revealed. It is often the case, however, that the outcome of an action is revealed after a substantial delay without sensory information about the chosen action. In everyday lives, feedbacks of a large number of actions are available not immediately, but with a delay on the timescale of seconds. Hence, we are frequently faced with the problem of attributing credit of an outcome to an action we committed seconds before. There are several candidate neural processes that can be used to solve this temporal credit assignment problem. First, synapses activated during action generation may be tagged biochemically, and their weights are modified by feedback signals^1,2^. Empirical studies have found evidence for molecular synaptic tagging in the striatum and cortex in rodents^3–6^ as well as in the insect brain^7^. Second, spike timing-dependent synaptic plasticity beyond millisecond timescale may associate two events that are separated in time at longer timescales^8^. In the hippocampus, a variant of spike timing-dependent synaptic plasticity rule (‘behavioral timescale’ synaptic plasticity rule) allows potentiation of synapses that are activated by presynaptic activity up to 2 s before^9^. Third, the information about an action may be maintained for a short period of time in the form of short-term synaptic weight changes^10,11^.

As another candidate mechanism, which is the subject of the present study, memory of the committed action may be maintained by persistent activity of a population of neurons^12^. This will be referred to as ‘active’ eligibility trace to contrast to the other candidate processes mentioned above (synaptic tagging and synaptic plasticity) which will be referred to as ‘silent’ eligibility traces. Active eligibility trace is a plausible neural process given that many brain structures maintain working memory in the form of persistent neuronal ensemble activity^13^. It has also been shown that many different brain areas in rats and monkeys maintain choice signals as persistent activity over multiple trials^12,14^. However, to the best of our knowledge, choice-related persistent activity has not been demonstrated in an experimental setting that requires temporal credit assignment. In almost all studies so far, sensory information about the chosen action was available at the time its outcome is revealed. In this regard, we have previously examined striatal neural activity in a task in which a choice must be remembered until its outcome is revealed for correct credit assignment; however, we failed to find persistent neuronal ensemble activity linking a choice and its outcome in the dorsal striatum^15^. In another study in which monkeys performed a task requiring temporal credit assignment across target-cue presentation and choice outcome, neural activity related to a target cue subsided following cue offset and then arose again at the time of outcome onset^16^. Hence, even though active eligibility trace is a plausible mechanism for temporal credit assignment, it is yet to be demonstrated empirically.

In the present study, we examined whether the medial PFC (mPFC) maintains eligibility trace in the form of persistent neuronal ensemble activity. Given its importance in working memory^17–20^ we reasoned that the mPFC may maintain short-term memory of a chosen action based on active discharges. Our results show that the mPFC neuronal population carries significant choice signals in the form of persistent activity during the time period between the animal’s choice and its outcome.

## Results

**Figure 1:**
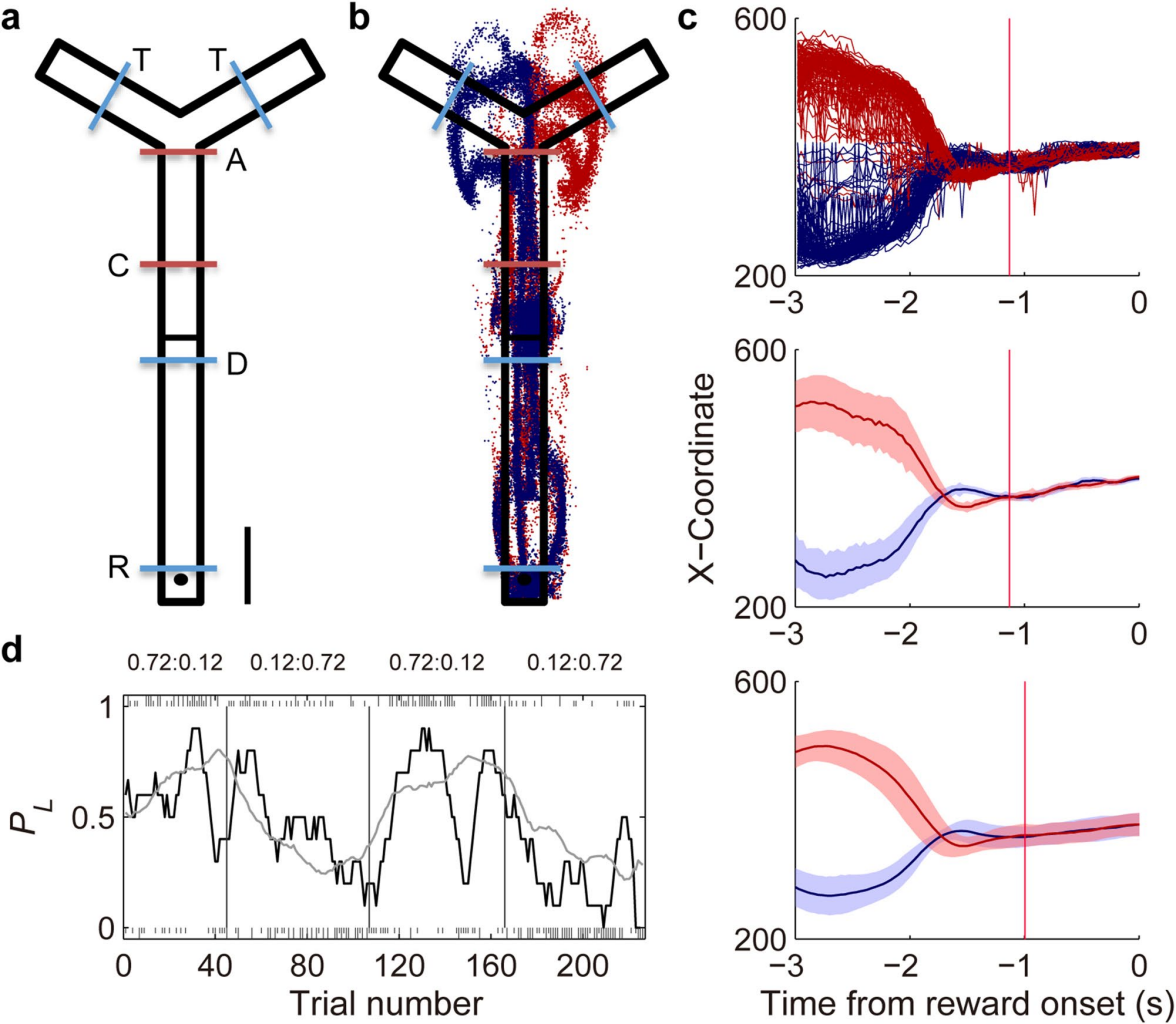
Behavior. (**a**) A modified T-maze. A trial began when the animal arrived at the delay point (D) from the proximal side of the central stem. Following a 2-s delay at the delay point, the animal was allowed to choose freely between two targets (T) and return to the reward location (R) where water reward was delivered probabilistically according to the animal’s choice. Blue dashed lines denote photobeam sensors. ‘A’ (approach) and ‘C’ (convergence) denote approximate spatial positions for the divergence (outbound) and convergence (inbound) of left and right target-associated movement trajectories, respectively. (**b**) Movement trajectories in a sample session. Blue, left choice; red, right choice. Each dot represent the animal’s head position at 33.3 ms time resolution. (**c**) Determination of the time of memory stage onset. The onset of memory stage was determined separately for each session according to the animal’s movement trajectories. The graphs show the time course of X-coordinates of animal’s position data during the 3-s time period before reward stage onset (time 0). The onset of memory stage (red vertical lines) was when the difference in X-coordinates of the left- and right-choice trajectories became statistically insignificant (*t*-test, *p* > 0.05) and maintained that way for at least three consecutive time points (100 ms). Top, X-coordinates of all return trajectories following left and right target choices (blue and red, respectively) during the sample session. Middle, mean (± SD across trials) X-coordinates of return trajectories for the same session. Bottom, mean X-coordinates of return trajectories averaged across sessions (± SD; n = 41). (**d**) Choice behavior during the sample recording session. Tick marks indicate the actual choices of the animal (upper, left choice; lower, right choice; long, rewarded; short, unrewarded). Vertical solid lines indicate block transitions. Numbers on the top denote mean reward probabilities following left and right choices in each block. The gray line shows the probability to choose the left target (*P*_*L*_) predicted by the Q-learning model. The black line shows the actual *P*_*L*_ in a moving average of 10 trials.

Three rats were trained in a dynamic two-armed bandit (TAB) task in an elevated Y-shaped maze as in our previous study^15^ (Fig. 1a). Each trial began when the rat arrived at the junction between the proximal and distal sections of the central stem ('D' in Fig. 1a; detected by a photobeam sensor) from the proximal side of the maze. Following a delay of 2 s, which was imposed by elevating a distal portion of the central stem, the rat was allowed to choose freely between two targets that were located at the distal ends of the maze. Each choice was associated with one of two different probabilities (12 or 72%) of water (30 μl) delivery that were constant within a block of 34–75 trials (59.2 ± 13.1; mean ± SD), but reversed across four blocks (total trials per session, 234.2 ± 27.0; mean ± SD) without any sensory cue. Hence, the rat had to discover changes in reward probabilities only by trial and error. The outcome of a choice was revealed not immediately after the choice, but when the rat returned to the proximal end of the maze (reward zone; 'R' in Fig. 1a; detected by a photobeam sensor) and waited for 0.5 s. The next trial began when the rat broke the photobeam at the junction between the proximal and distal sections of the central stem ('D' in Fig. 1a).

The central stem of the maze was narrow (5 cm) so that the rat’s return trajectories from the left and right targets to the reward zone converged on the central stem (Fig. 1b). As a consequence, no explicit sensory cue on the animal’s chosen target was available at the time the outcome was revealed (0.5 s following the rat’s arrival at the reward zone). Hence, in this task, the animal was required to maintain eligibility trace during the time period between trajectory convergence and outcome onset (‘memory’ stage) and combine this information with outcome information for correct credit assignment. The onset of memory stage was determined separately for each session (Fig. 1c). The mean (± SD) duration of the memory stage was 1.0 ± 0.4 s.

The rats were over-trained in the task (99, 40 and 95 sessions) before unit recording began. Figure 1d shows the rat’s performance in the task in a sample recording session. As shown in this example, the rat initially chose the lower-probability (12%) target more frequently after block transition. However, after ~ 10–20 trials since block transition, the rat switched its preferential choice to the higher-probability (72%) target. Overall, during the recording sessions, the rats chose the higher-probability target in 62.6 ± 7.7% (mean ± SD across sessions) of trials and rewarded in 50.5 ± 5.5% of trials. During the first 10 trials after block transition, the rats chose the higher-probability target in 41.7 ± 13.3% of trials and were rewarded in 37.2 ± 10.8% of trials; during the last 20 trials of a block, they chose the higher-probability target in 75.4 ± 10.7% of trials and were rewarded in 57.6 ± 8.1% of trials (only blocks 2–4 were analyzed). The rats showed slight choice biases (proportion of left choices, 51.4, 50.8 and 47.9%).

**Figure 2:**
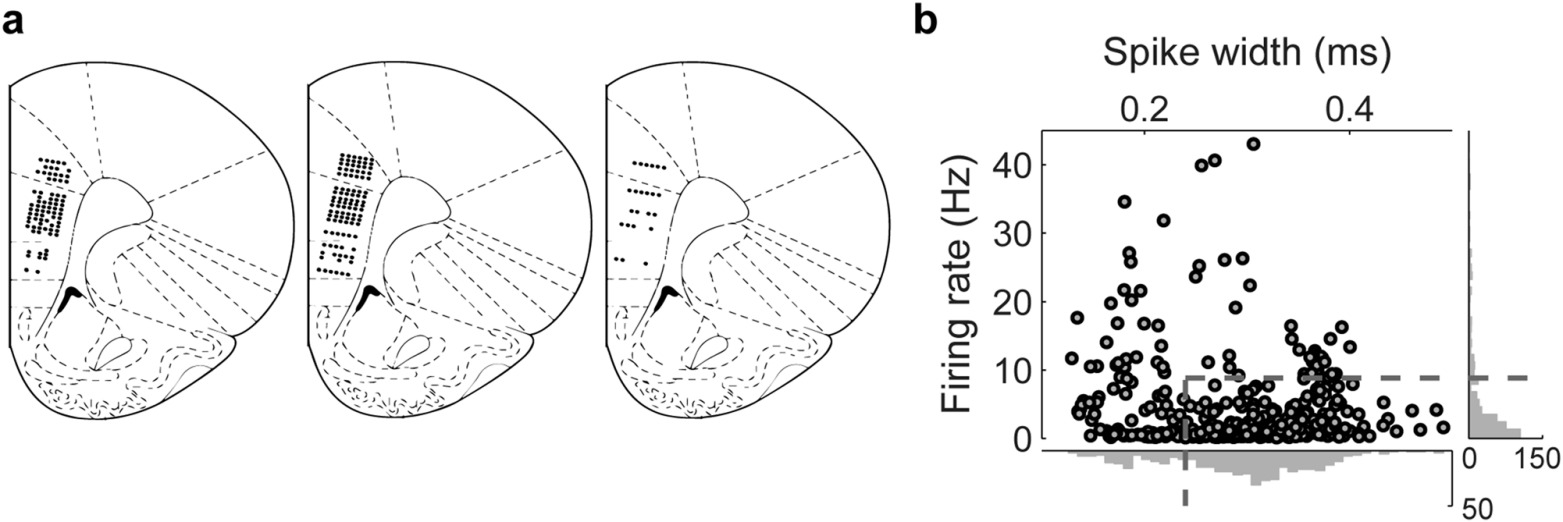
Recording sites and unit classification. (**a**) Single units were recorded from the anterior cingulate cortex (ACC), prelimbic cortex (PLC), and infralimbic cortex (ILC). The diagrams are coronal sections of three rat brains at 3.24 mm anterior to bregma. Each diagram represents one brain and each circle represents one recording site that was estimated based on histology and electrode advancement history. Units recorded near the borders were excluded. Modified with permission from ref.^34^. (**b**) The recorded units were classified into putative pyramidal cells and putative interneurons based on mean firing rates and spike widths (filtered spike waveforms; peak-to-valley). Those units with mean firing rates < 8.83 Hz and spike widths > 0.24 ms were classified as putative pyramidal cells and the rest were classified as putative interneurons.

We recorded total 446 single units from the anterior cingulate cortex (ACC), prelimbic cortex (PLC) and infralimbic cortex (ILC) in the right hemisphere in three rats (excluding those units recorded near the borders between these structures) performing the dynamic TAB task over 12–21 days (Fig. 2a). The recorded units were classified into putative pyramidal neurons (n = 298) and putative interneurons (n = 148) based on mean discharge rates and spike widths (Fig. 2b). Only putative pyramidal neurons were included in the analysis (78 ACC, 160 PLC and 60 ILC units; mean discharges rates, 2.0 ± 1.7, 1.8 ± 1.8 and 1.9 ± 2.0 Hz, respectively; mean ± SD).

**Figure 3:**
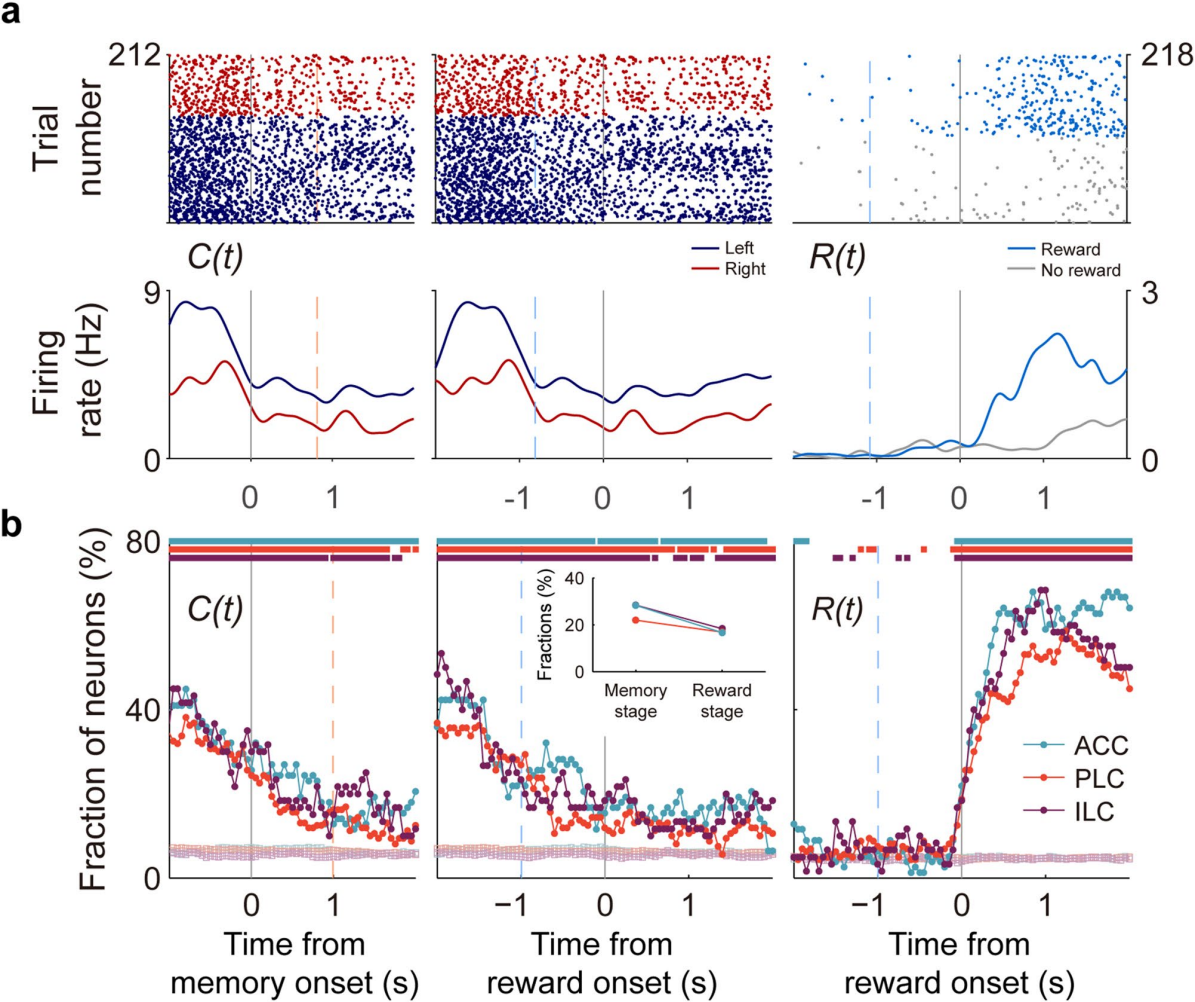
Neural activity related to choice and outcome. (**a**) Sample neurons coding the animal’s choice [*C(t*)] during the memory stage (left) or choice outcome [*R(t*)] during the reward stage (right). Orange and light blue dashed lines, onset times of the reward and memory stages, respectively. (**b**) Population data showing temporal profiles of choice and outcome signals during the memory and reward stages. Shown are fractions of neurons significantly responsive to a given variable (500-ms sliding window advanced in 50-ms steps; n = 78 ACC, 160 PLC, and 60 ILC units). Cyan, ACC; orange, PLC; purple, ILC. Filled circles, actual data; empty square, trial-shifted data (control). Light orange and light blue dashed lines, mean onset times (averaged across all sessions) of the reward and memory stages, respectively. Colored squares/bars on top, significant differences (permutation test, *p* < 0.05) between the original and trial-shuffled data. Inset, fractions of choice-coding neurons during the memory and reward stages (first 1 s each).

We first examined whether mPFC neurons carry active eligibility trace signals during the memory stage and whether two signals necessary for temporal credit assignment, namely eligibility trace and outcome signals, coexist in the mPFC during the reward stage. For this, we examined temporal profiles of mPFC neuronal activity related to the animal's choice and its outcome. Neural activity was analyzed in a 500-ms moving window that was advanced in 50-ms steps and temporally aligned to memory stage as well as reward stage onsets. To control for potential confounding influences of signals related to the animal's choice and its outcome in the previous trials^12,14^, we ran a multiple regression analysis that included the animal's choice and its outcome in the current and two previous trials as explanatory variables (Eq. 4). Both eligibility trace- and outcome-coding neurons were found in the mPFC as shown by sample neurons in Fig. 3a. Figure 3b shows fractions of neurons significantly responsive to the animal's choice [*C(t*)] or outcome [*R(t*)] in the current trial. As shown, neuronal populations in all three subregions carried significant choice signals (permutation test using trial-shifted data; *p* < 0.05) throughout the memory stage and also during the reward stage. Overall, the return time (time between photobeam activations at ‘T’ and ‘R’ in Fig. 1a) was slightly, but significantly shorter following high compared to low reward-probability-target choices (3.6 ± 1.5 and 3.7 ± 1.7 s, respectively; mean ± SD; *t*-test, *t*(9642) = 3.407; *p* < 0.01). Similar results were obtained when we performed the same analysis excluding those sessions with significantly (*t*-test, *p* < 0.05) different return times between high and low reward-probability-target choices (n = 9 out of 41 sessions; 22.0%; see Supplementary Fig. S1), indicating that the persistent choice signals are not because of positional variations associated with different reward probabilities.

The choice signals became progressively weaker since the memory stage onset in all three subregions (Fig. 3b). Relative strength of choice signals did not vary significantly across the subregions in the memory stage (first 1 s or the entire memory stage in those sessions with memory stage < 1 s; χ^2^-test, χ^2^ = 0.445, *p* = 0.45) or reward stage (first 1 s; χ^2^ = 0.080, *p* = 0.96; Fig. 3b). Reward signals were weak before reward stage onset, but rose rapidly since the reward stage onset so that significant choice and outcome signals coexisted during the reward stage in all three subregions. These results indicate that the mPFC maintains active eligibility trace during the memory stage and carries concurrent eligibility trace and outcome signals during the outcome stage.

To test whether choice and outcome signals are conjunctively encoded in the mPFC, we examined whether mPFC neurons are more likely to encode both choice and outcome signals than expected by chance. Of 78 ACC neurons, 13 and 48 were significantly responsive to choice and outcome, respectively, during the first 1-s time period since reward stage onset (the time the outcome was revealed; 0.5 s following the rat’s arrival at the reward zone), and, of these, 11 were significantly responsive to both choice and outcome. There was a trend for ACC neurons to encode both choice and outcome more than expected by chance (χ^2^-test, χ^2^ = 3.510, *p* = 0.06). Analyzing the same time period, of 160 PLC neurons, 27 and 71 were significantly responsive to choice and outcome, respectively, and 19 were significantly responsive to both choice and outcome; of 60 ILC neurons, 11 and 32 were significantly responsive to choice and outcome, respectively, and 10 were significantly responsive to both choice and outcome. Both PLC and ILC neurons were significantly more likely to encode both choice and outcome than expected by chance (χ^2^-test, PLC, χ^2^ = 8.892, *p* < 0.01; ILC, χ^2^ = 7.641, *p* < 0.01). These results indicate that choice and outcome signals are conjunctively combined in the mPFC during the reward stage.

**Figure 4:**
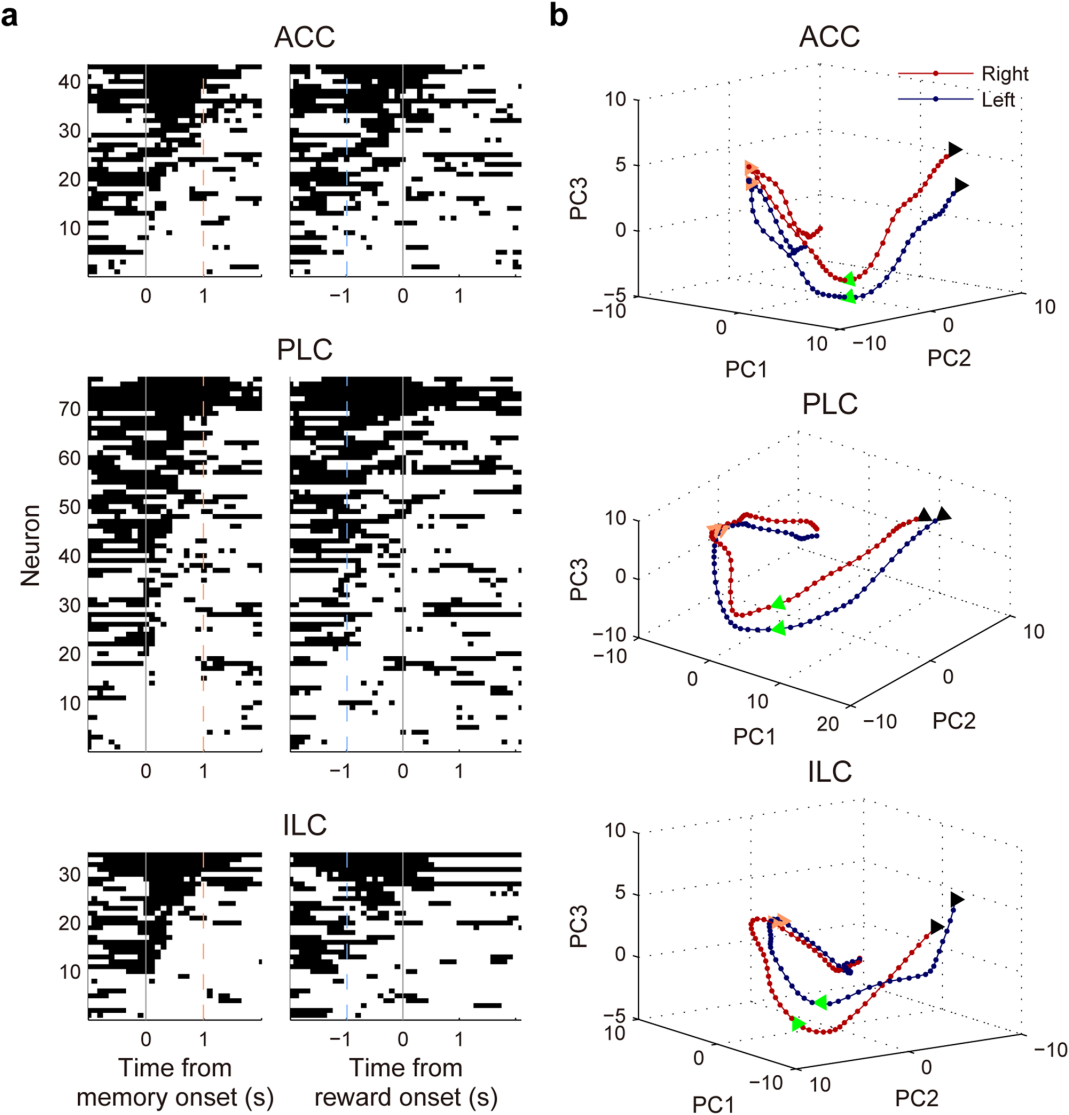
Dynamics of choice-related neural activity during memory stage. (**a**) Shown are those mPFC neurons that are significantly responsive to the animal’s target choice during at least one analysis window (500 ms, advanced in 100-ms steps) during the memory stage. Each horizontal line segment indicates significantly different (*t*-test, *p* < 0.05) neuronal activity between left- and right-choice trials in that analysis time window. Neurons were ordered according to the total duration of significant choice-selective responses. Neuronal activity was aligned to the onset of memory (left) or reward (right) stage. Light orange and light blue dashed lines, mean onset times (averaged across all sessions) of the reward and memory stages, respectively. (**b**) Trajectories of the three principal components of the mPFC neural populations associated with the left (blue) and right (red) target choices. Filled circles denote 50-ms time steps. Black triangles, 1 s before memory stage onset; green triangles, onset of memory stage; orange triangles, mean onset time (averaged across all sessions) of the reward stage.

We then examined whether individual mPFC neurons carry eligibility trace signals in the form of persistent activity. For this, we examined temporal profiles of individual neuronal responses to the animal’s target choice during the memory stage using a moving-window analysis (500 ms, advanced in 100-ms steps). As shown in Fig. 4a, the majority of mPFC neurons conveyed eligibility trace signals only during certain phases of the memory stage; only a small fraction (ACC, 5 out of 78, 6.4%; PLC, 7 out of 160, 4.4%; ILC, 5 out of 60, 8.3%) showed significant choice-related activity throughout the entire memory stage. Consequently, neuronal ensemble activity went through dynamic changes during the memory stage (Fig. 4b). These results indicate that active eligibility trace signals are maintained by dynamically changing, rather than stably maintained, mPFC neuronal population activity in our task.

**Figure 5:**
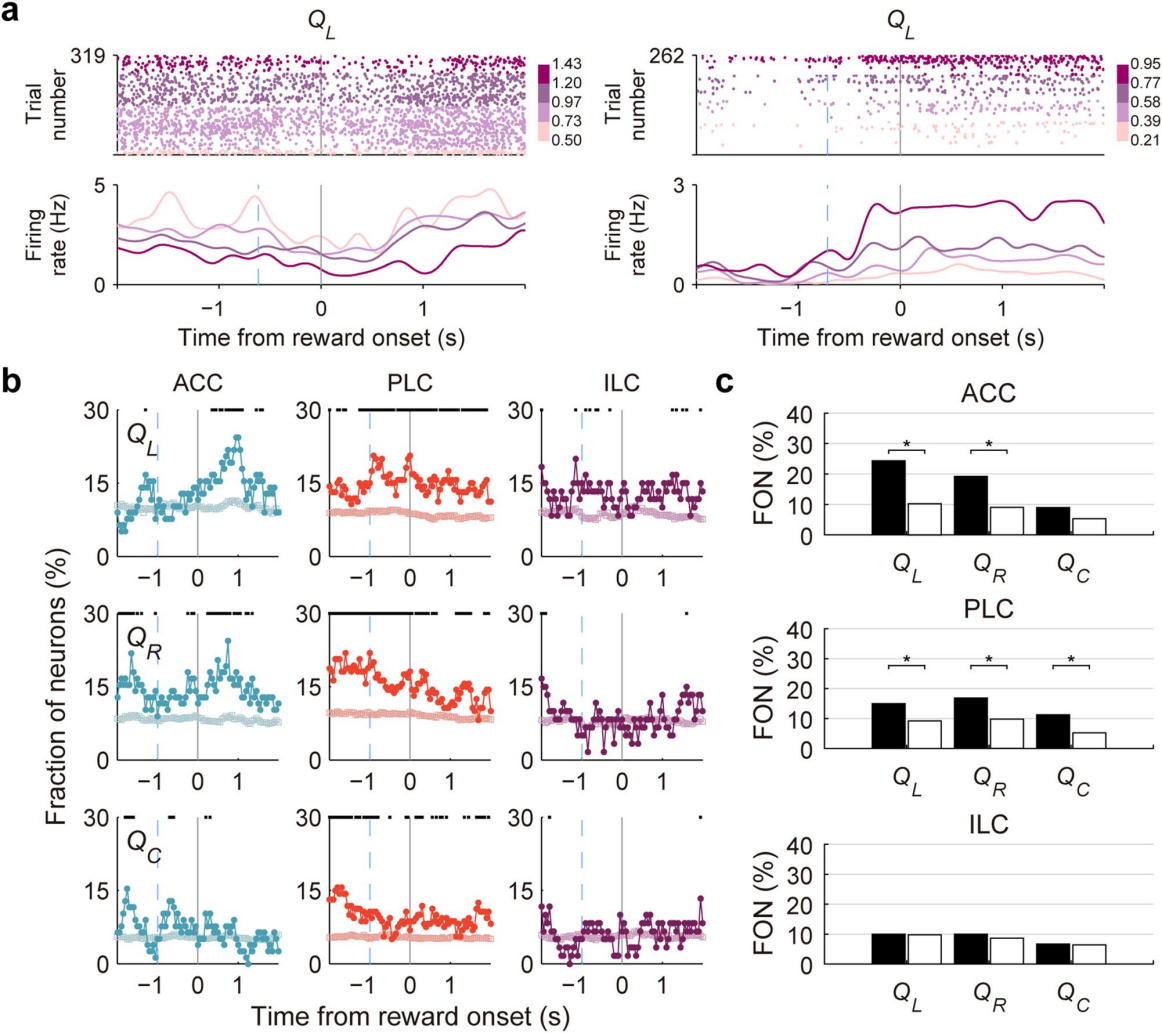
Neural activity related to action value and chosen value. (**a**) Sample neurons coding left action value (*Q*_*L*_). Trials were grouped into quartiles of left action value. Light blue dashed line, onset time of memory stage for the corresponding session. (**b**) Population data showing temporal profiles of neural signals for left action value (*Q*_*L*_), right action value (*Q*_*R*_), and chosen value (*Q*_*C*_) around the onset of reward stage (time 0). Shown are fractions of neurons significantly responsive to a given variable (500-ms sliding window advanced in 50-ms steps). Light blue dashed line, mean onset time of memory stage. Cyan, ACC; orange, PLC; purple, ILC. Filled circles, actual data; empty square, trial-shifted data (control). Black squares/bars on top, significant differences (permutation test, *p* < 0.05) between the original and trial-shuffled data. (**c**) Action value and chosen value signals during the first 1 s of the reward stage. Filled bars, actual data; empty bars, trial-shifted data (control). FON, fraction of neurons. *Significant difference between the actual and trial-shifted data (*p* < 0.05, permutation test).

Choice and outcome signals must be combined with value signals in order for the animal to update expected outcomes associated with two target choices. In our previous studies using conventional dynamic foraging tasks, signals for chosen value (value of the chosen action in a given trial; i.e., left action value in left-choice trials and right action value in right-choice trials) were elevated as the animals approached the target locations, so that they were combined with outcome signals at the time choice outcome was revealed in numerous brain areas^21–25^. Chosen value and outcome signals would be sufficient to compute reward prediction error and update value of the chosen target in a given trial. However, in our previous study in the striatum that used the same task as the present one, we failed to observe elevation of chosen value signals at the time of choice outcome^15^. Instead, action value signals were elevated in the dorsolateral (but not dorsomedial) striatum. Given that choice and outcome signals were also significant at the time of choice outcome, these results suggest that the dorsolateral striatum my update value of the chosen target by combining signals for action value, choice, and outcome. To examine neural signals for action value and chosen value in the mPFC, we used a regression model that included left action value, right action value, and chosen value along with the animal's current choice and its outcome (Eq. 5; sample neurons coding action value are shown in Fig. 5a). Action values were computed using the hybrid model used in our previous study^15^ (Q-learning model combined with win-stay and lose-switch terms; Eqs. (1)–(3)) which well predicted the animal's actual choice behavior (Fig. 1d). As in the striatum, we failed to find elevation of chosen value signals at the onset of reward stage in all three regions of the mPFC. We also found significant action value signals (permutation test using trial-shifted data; *p* < 0.05) in the ACC and PLC (Fig. 5a,b). Signals for left and right action values were significantly above chance level during the first 1-s time period since reward stage onset in the ACC (permutation test, *p* < 0.01 for both) and PLC (*p* = 0.01 and < 0.01, respectively), but not in the ILC (*p* = 0.56 and 0.36, respectively) (Fig. 5b,c). Chosen value signals were significantly above chance level during the same time period in the PLC (*p* < 0.01), but not in the ACC (*p* = 0.11) or ILC (*p* = 0.49). In addition, in the ACC, chosen value signals were significantly weaker than left action value signals (χ^2^-test, χ^2^ = 6.646, *p* = 0.01). These results indicate that chosen value signals are not elevated in the mPFC at the time choice outcome is revealed in our task. They also indicate that all the signals necessary for updating action value, namely choice, outcome, and action value signals converge in the ACC and PLC during the early reward stage.

## Discussion

We examined whether the mPFC neuronal population carries memory signals for a chosen action in the form of persistent activity in a task that requires credit assignment across a temporal gap of ~ 1 s. We found that all subregions of the mPFC (ACC, PLC and ILC) maintain memory signals for a chosen action in the form of persistent ensemble activity during the time period between the animal's choice of action and its outcome. We also found that, unlike in conventional dynamic foraging tasks, chosen value signals are not elevated at the time of outcome onset. Instead, action value signals converged with choice and outcome signals in the ACC and PLC. These results show that all subregions of the mPFC actively maintain eligibility trace signals, and that they are combined with action value and outcome signals in the ACC and PLC when the animal’s choice outcome is revealed.

Our results provide evidence for active eligibility trace in the mPFC in the timescale of seconds. However, it remains to be determined whether such active eligibility trace actually contributes to assigning credit of the experienced outcome to the chosen action. Even in the case it does, it is unknown whether the mPFC relies solely on active eligibility trace to solve the temporal credit assignment problem. Other neural processes than active eligibility trace, such as synaptic tagging^1,2^, may also contribute to solving the temporal credit assignment problem in the mPFC. Also unknown is whether and how different brain structures rely on different neural processes to solve the temporal credit assignment problem. We failed to obtain evidence for active eligibility trace in the dorsal striatum in the same task as used in the present study^15^. This might be an indication that the mPFC plays a more important role than the striatum in temporal credit assignment in the current task (c.f., Ref.^26^). Alternatively, the mPFC and striatum may rely on different neural processes for temporal credit assignment. In this regard, previous studies have shown evidence for synaptic tagging in the striatum^3–5^. Note that experimental settings vary widely across studies on the neural basis of temporal credit assignment. A given brain structure may rely on different neural processes to solve temporal credit assignment depending on task requirement such as the length of a temporal gap between an action and its outcome^27^. Clearly, further studies are needed to clarify whether and how different brain structures utilize different neural processes to solve temporal credit assignment problem in diverse behavioral settings.

Assuming that the mPFC relies on active eligibility trace to solve the temporal credit assignment problem in the present task, it is unclear how the mPFC ascribes credit of a choice outcome to neuronal ensemble activity representing a specific choice. Even though the mPFC neuronal population maintained choice signals throughout the memory stage, only a small fraction of individual mPFC neurons showed persistent choice-related activity throughout the memory stage. In other words, choice signals were maintained in the form of dynamically changing neuronal ensemble activity during the memory stage so that neuronal ensemble activity at the time of outcome onset differed from that at the time of action selection. It is currently unclear how a choice outcome might alter mPFC neural network so that it affects neural representation of the chosen action in a way to guide future choices. In fact, this is a problem not limited to temporal credit assignment. The rodent mPFC, which plays a crucial role in diverse working memory tasks^28^, conveys working memory signals in the form of dynamically-changing, rather than stably-maintained, neuronal ensemble activity^17,18,20^. Readout of such dynamically-changing neuronal ensemble activity would be trivial if the duration of delay is fixed; a specific ensemble activity pattern at the end of delay can be matched to a specific sensory cue presented before delay. However, working memory signals are conveyed by dynamically-changing mPFC neuronal ensemble activity even when delay duration is not fixed, such as when delay duration is randomized across trials^29^. We also found the maintenance of previous choice signals in the form of dynamically-changing neuronal ensemble activity during the time period that varied across trials in the dorsomedial striatum^22^. Together, these results raise the possibility that the brain might be equipped with a mechanism for reading out signals reliably from dynamically-changing neuronal ensemble activity. Further studies are needed to elucidate whether this is the case and, if so, how such readout may be implemented.

A large body of studies have shown that chosen value signals arise at the time of outcome onset in multiple regions of the brain including the mPFC in behavioral tasks in which sensory information is available at the time of outcome onset^14,23,30^. These results suggest that these brain areas might update action value by combining chosen value and outcome signals. However, we found that action value signals are weak in the mPFC at the time of outcome onset in the present task. In our previous study that used the same task as the present one, we showed that chosen value signals are weak and, instead, action value signals are elevated during the reward stage in the dorsolateral striatum^15^. These results suggest that dynamics of neural signals related to evaluating choice outcome might differ drastically depending on the requirement for temporal credit assignment in the mPFC and dorsal striatum. These brain structures may rely on action value rather than chosen value signals in updating value of the chosen action when sensory information on the chosen action is not available at the time of outcome. It remains to be determined whether this is a common rule that can be applied to other brain structures as well.

## Methods

### Animals

Three young male Long-Evans rats (~ 11–15 weeks old, 300-360 g) were individually housed in a colony room and initially allowed free access to food and water. They were then gradually deprived of water with free access to food with extensive handling for 7 days. Their body weights were maintained at > 80% ad libitum throughout the experiments. Experiments were performed in the dark phase of a 12-h light/dark cycle. All experiments were performed in accordance with protocols approved by the directives of the Animal Care and Use Committee of the Korea Advanced Institute of Science and Technology (Approval Number: KA2018-08).

### Behavioral task

The rats performed a dynamic TAB task in a Y-shaped maze that was made of black acrylic (Fig. 1a). The central stem was 70 cm long and each of the two distal arms was 20 cm long. The width of the track was 5 cm and there was 5-cm wall along the track except around the junction between the central and distal arms to allow turning behavior. Each trial began when the rat arrived at the junction between the lower and upper segments of the central stem from the reward zone (proximal end of the central stem) and broke a photobeam (delay position; ‘D’ in Fig. 1a). The upper segment of the central arm (25 cm long) was elevated before each trial onset to prevent the animal from navigating forward. The upper segment of the central stem was lowered 2 s following trial onset (i.e., 2-s delay was imposed at the outset of each trial) allowing the rat to navigate forward and choose freely between two targets. The rat’s choice of a target was detected by two photobeam sensors that were located in the middle of the distal arms (‘T’ in Fig. 1a). Activation of the left or right target photobeam sensor triggered an auditory cue (left, 4.3 kHz; right, 4.5 kHz) until the photobeam-breaking ended. The outcome of the rat’s choice was revealed when the rat returned to the reward zone and waited for 0.5 s (reward stage onset). The rat’s arrival at the reward zone was detected by a photobeam sensor (‘R’ in Fig. 1a). Positive (30 µl water delivery) and negative (no reward delivery) outcomes were signaled by two different sound cues (100 ms; 1 and 9 kHz for positive and negative outcomes, respectively in 2 rats and for negative and positive outcomes, respectively in 1 rat) that were delivered at the reward stage onset. The next trial began as soon as the animal arrived at the delay position and broke the photobeam. No time limit was set for the rats to return to the delay position from the reward zone or to make a choice since delay offset. During the recording sessions, the rat stayed 9.1 ± 5.6 and 3.9 ± 5.0 s (mean ± SD) since the outcome onset in the reward zone in rewarded and unrewarded trials, respectively, and arrived at either target arm in 0.7 ± 0.9 s (mean ± SD) since the delay offset. Even though the rats approached either target arm in all trials, they failed to activate a target photobeam sensor in some trials (n = 184; 1.9% of total trials) that were excluded from the analysis. Each session consisted of four blocks of 34–75 trials (59.3 ± 13.1, mean ± SD; first block, 40 plus a randomly chosen number between 0 and 20; the other blocks, 45 plus a randomly chosen number between 0 and 30). One of two reward probability configurations (left:right, 0.72:0.12 and 0.12:0.72) was chosen randomly for the first block and it was reversed during each block transition (0.72:0.12 to 0.12:0.72 or 0.12:0.72 to 0.72:0.12). The rats were trained until they chose the high reward-probability target > 80% in the steady state (last 10 trials in each block) before unit recording began.

### Determination of memory stage onset

The onset of the memory stage was determined separately for each session. We temporally aligned the rat’s X-position data to the time point 3 s prior to the reward stage onset. The time point when the difference in X-coordinates of the left- and right-choice trajectories become statistically insignificant (*t*-test, *p* > 0.05) and maintained that way for at least three time points (100 ms) was marked as the onset of the memory stage (Fig. 1c).

### Reinforcement learning model

We used the same hybrid model we used in our previous study^15^ to calculate trial-by-trial action values. This model includes simple reinforcement learning and win-stay-lose-switch terms. For the chosen action ‘*a*’ in the *t*-th trial, the action value *q*_*a*_*(t* + *1)* was updated as follows:

1 $$q_{a} (t + 1) \, = \, q_{a} (t) \, + \alpha \cdot (R(t) - q_{a} (t)),$$

where *α* is the learning rate and *R(t)* is the reward (i.e., trial outcome; 1 if rewarded and 0 otherwise) in the *t*-th trial. The total action value [*Q*_*a*_*(t* + *1)*], that includes the action value [*q*_*a*_*(t* + *1)*] and the win-stay-lose-switch term, were calculated as follows:

2 $$\begin{aligned} & {\text{if}}\,R(t) = { 1} \\ & \quad Q_{a} \left( {t + 1} \right) \, = \, q_{a} \left( {t + 1} \right) \, + \, a_{WS} \\ & {\text{else}} \\ & \quad Q_{a} \left( {t + 1} \right) \, = \, q_{a} \left( {t + 1} \right) \, + \, a_{LS} , \\ \end{aligned}$$

where *a*_*WS*_ and *a*_*LS*_ are the win-stay and lose-switch terms, respectively. The action value for the unchosen action did not change. The probability for selecting the left goal ${P}_{L}(t)$ was defined as follows

3 $${P}_{L}(t) = \frac{1}{{1 + {\exp}\left( { - \beta \left( {Q_{L} \left( t \right) - Q_{R} \left( t \right) - \gamma } \right)} \right) }},$$

where *β* is the inverse temperature which defines the degree of exploration during the action selection and $\gamma$ is a term for the bias in selecting the right target. The model parameters were estimated for each rat using the maximum likelihood procedure^31^.

### Unit recording

Single units were recorded with tetrodes from the dorsal ACC, PLC and ILC (Fig. 2a). Fifteen tetrodes were implanted targeting the right mPFC (3.24 mm anterior and 1.2 mm lateral to bregma; 0.7 mm ventral from the brain surface) at an angle 5° toward the midline under deep anesthesia with isoflurane (2.5–3.0% [v/v] in 100% oxygen). The tetrodes were gradually lowered into the ACC over 7–15 days. Once the recording began, the tetrodes were advanced by 35–50 μm after a daily recording session. Unit signals were amplified 5000–10000× , band pass-filtered between 600–6000 Hz, digitized at 34 kHz, and stored on a personal computer via a Cheetah data acquisition system (Neuralynx, Bozeman, MO, USA). Unit signals were recorded with the animals placed on a custom-made pedestal for 5 min before and after each recording session to test the stability of recorded units. The head position of the animals were recorded at 30 Hz by tracking an array of light emitting diodes located on the headstage. When the recording procedures were completed, small marking lesions were made by passing an electrolytic current (30 µA, 20 s, cathodal) through one channel of each tetrode at the end of the final recording, and their locations were verified histologically as previously described^32^. Unit recording locations were determined based on the locations of marking lesions and tetrode advancement history. Those units that were determined to be near the border between the secondary motor cortex and ACC, between the ACC and PLC, or between the PLC and ILC were excluded from the analysis (Fig. 2a).

### Isolation and classification of units

Single units were isolated by manually clustering various spike waveform parameters using MClust software (v3.5; A. R. Redish). The identity of a unit signal was determined based on the clustering pattern of spike waveform parameters, averaged spike waveforms, baseline discharge frequencies, autocorrelograms and interspike interval histograms^22^. Only those clusters with no inter-spike interval < 3 ms, L-ratio < 0.2, isolation distance > 15^33^, and the number of spikes > 500 were included the analysis. The recorded units were classified into putative pyramidal neurons and putative interneurons based on average firing rates and spike widths. Those units with average firing rates < 8.83 Hz and filtered spike waveform widths > 240 μs were classified as putative pyramidal neurons (n = 298) and the rest were classified as putative interneurons (n = 148; Fig. 2b). Only putative pyramidal neurons were included in the analysis.

### Multiple regression analysis

Neural activity related to the animal’s choice and its outcome was examined using the following regression model:

4 $$\begin{aligned} S\left( t \right) & = a_{0} + a_{1} C\left( t \right) + a_{2} C\left( {t - 1} \right) + a_{3} C\left( {t - 2} \right) + a_{4} R\left( t \right) + a_{5} R\left( {t - 1} \right) + a_{6} R\left( {t - 2} \right) \\ & \quad + a_{7} X\left( t \right) + a_{8} X\left( {t - 1} \right) + a_{9} X\left( {t - 2} \right) + \varepsilon \left( t \right), \\ \end{aligned}$$

where ${S}\left({t}\right)$ indicates spike discharge rate in a given analysis time window, ${C}\left({t}\right)$, ${R}\left({t}\right)$, and ${X}\left({t}\right)$ represent the animal’s choice (left or right; dummy variable, − 1 or 1), its outcome (dummy variable, − 1 or 1), and their interaction, respectively, in trial *t*. ${a}_{0}$~${a}_{9}$ are regression coefficients and $\varepsilon \left(t\right)$ is the error term.

Neural activity related to action value and chosen value was examined with the following model:

5 $$S\left( t \right)= {a}_{0}+{a}_{1}C\left(t\right)+ {a}_{2}R\left(t\right) +{a}_{3}X\left(t\right) + {a}_{4}{Q}_{L}\left(t\right)+{a}_{5}{Q}_{R}\left(t\right)+{a}_{6}{Q}_{C}\left(t\right)+A\left(t\right)+ \varepsilon \left(t\right),$$

where ${Q}_{L}(t)$ and ${Q}_{R}(t)$ denote action values for choosing the left and right targets, respectively, and ${Q}_{C}(t)$ indicates chosen value (action value of the chosen action in a given trial), that were estimated using the hybrid model, in trial *t*. *A(t)* is a set of autoregressive terms consisting of spike discharge rates during the same analysis time window in the previous three trials as the following:

6 $$A\left(t\right)= {a}_{7}S\left(t-1\right)+ {a}_{8}S\left(t-2\right) {+ a}_{9}S\left(t-3\right).$$

Action values are correlated across trials because they are updated incrementally according to the animal’s choice outcomes. If neural spikes are also correlated across trials, they may appear as encoding action value even though the source of spike serial correlation is unrelated to action value. We included *A(t)* in the regression model to prevent such slowly drifting neural activity from inflating action value signals.

### Statistical tests

Sample sizes were determined based on the sample sizes in our previous study^15^ in which dorsal striatal neural activity was examined in the same behavioral task as used in the present study. Student’s *t*-tests were used to determine the significance of a regression coefficient and the difference between left- and right-choice trajectories. Permutation tests were used to determine significance of neural signals for a given variable. We circularly shifted trials of spike count data by a random number (with the constraint that the minimum difference of trial number between the original and shifted data is > 10) and assessed how many neurons were significantly responsive to a variable of interest. This was repeated 100 times, and the *p* value for a given variable was determined by the frequency in which the number of neurons significantly responsive to a given variable was exceeded by that obtained after trial shuffling. χ^2^-tests were used to test whether choice and outcome signals are encoded conjunctively. All tests were two-tailed, and a *p* value < 0.05 was used as the criterion for a significant statistical difference. Results are expressed as mean ± SD.

## Supplementary information

Supplementary Figure S1.

## Data Availability

Raw data and code to reproduce this work is archived at: https://www.dropbox.com/sh/ihjk0bunf3jmmnl/AAAN77uo2bbP3kozcL06-_x7a?dl=0.
