# Supplementary material for: Active maintenance of eligibility trace in rodent prefrontal cortex
Source: Sci Rep. 2020 Nov 2;10:18860. doi: 10.1038/s41598-020-75820-0 (PMC7608665; doi:10.1038/s41598-020-75820-0)
Supplement: Supplementary file 1 — Supplementary Figure S1. [file 41598_2020_75820_MOESM1_ESM.pdf]

Supplementary Information

Active maintenance of eligibility trace in rodent prefrontal cortex

Dong-Hyun Lim, Young Ju Yoon, Eunsil Her, Suehee Huh & Min Whan Jung

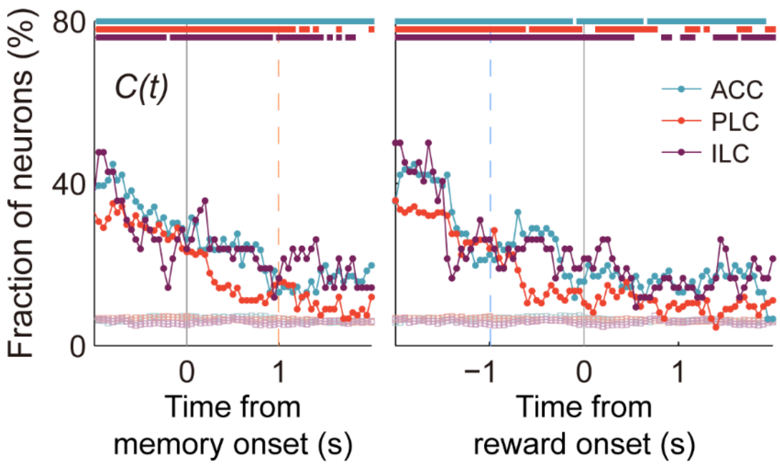

Figure S1. Choice-related neural activity excluding sessions with different return times. Population data showing temporal profiles of choice signals during the memory and reward stages. Only those sessions without significantly (*t*-test, $p < 0.05$) different return times between high and low reward-probability-target choices (n = 32 out of 41 sessions; n = 76 ACC, 134 PLC, and 42 ILC units) were included in the analysis. The same format as in Fig. 3b.
